# Musashi2 promotes the development and progression of pancreatic cancer by down-regulating Numb protein

**DOI:** 10.18632/oncotarget.8736

**Published:** 2016-04-15

**Authors:** Weiwei Sheng, Ming Dong, Chuanping Chen, Yang Li, Qingfeng Liu, Qi Dong

**Affiliations:** ^1^ Department of General Surgery, Gastrointestinal Surgery, The First Hospital, China Medical University, Shenyang, 110001, China; ^2^ Department of Clinical Laboratory, The Sixth Peoples’ Hospital of Shenyang City, 110003, China; ^3^ Department of Cell Biology, China Medical University, Shenyang, 110001, China; ^4^ Department of General Surgery, The Peoples’ Hospital of Liaoning Province, Shenyang, 110015, China

**Keywords:** Musashi2, Numb, invasion and metastasis, pancreatic cancer

## Abstract

Musashi2-Numb interaction plays a vital role in the progression of myeloid leukemia. However, its potential role in solid cancers has rarely been reported. We investigated the coordinate function of Musashi2-Numb in the development of pancreatic cancer (PC) in vitro and vivo. Both Musashi2 protein and mRNA levels were higher in PC tissues than that in paired normal pancreas (*P*<0.05). Musashi2 overexpression and Numb positive expression were positively and negatively associated with tumor size and UICC stage, respectively (*P*<0.05). Multivariate analysis identified Musashi2 and Numb as adverse and favorable independent indicators for the survival of PC patients. Moreover, patients with high Musashi2 expression combining with negative Numb expression had a significantly worse overall survival (*P*=0.001). The negative relationship between Musashi2 and Numb was found at both PC tissue and cell levels. These two endogenous proteins can be co-immunoprecipitated from PC cell lines, and Musashi2 silence up-regulated Numb protein in vitro and vivo. Meanwhile, its silence decreased cell invasion and migration in vitro and inhibited the growth of subcutaneous tumors and the frequency of liver metastasis in vivo. However, Numb knockdown significantly reversed the decrease of cell invasion and migration induced by Musashi2 silence. Musashi2 promotes the development and progression of pancreatic cancer by down-regulating Numb protein. The interaction of Musashi2-Numb plays a significant role in the development and progression of PC.

## INTRODUCTION

Pancreatic cancer (PC) remains one of the most aggressive malignant tumors with high recurrence and much poor prognosis. The lethality of PC is mainly attributable to its strong local invasion and high distant metastasis (25-50%) [1]. It is urgent to identify the new molecular biomarkers in predicting the aggressive biology of PC.

Musashi (MSI), as an evolutionarily conserved family of RNA-binding proteins, comprises two related proteins (MSI1 and MSI2) and acts as a translational repressor [2]. It is originally identified in stem and early progenitor cells [3, 4] and plays a critical role in asymmetric cell division, stem cell function and cell fate determination in various somatic tissues [5]. Mounting evidence shows that MSI2 is an important proliferation/differentiation modulator in normal hematopoietic, embryonic stem cells (ESC) [3, 6] and myeloid leukemia [7, 8]. Loss of MSI2 has been reported to block the self renewal of ESC and impair the development and propagation of blast crisis chronic myeloid leukemia (CML) [6, 8]. Recent studies show a deregulation of MSI2 in some solid tumors, such as hepatocellular, gastric and brain cancers [9–11]. Moreover, MSI2 knockdown decreases the growth of glioblastoma and medulloblastoma cells and inhibits cell invasion in hepatocellular carcinoma [9, 11]. However, its potential role in the development and progression of PC has been not reported yet.

Numb is originally discovered as the first intrinsic molecular determinant of cell fate in drosophila sensory organ development [12], and is lately identified as a significant regulator in cancers [13]. Our previous study showed a suppressive role of Numb in cell invasion and migration of PC [14], but the corresponding molecular mechanisms have not been fully understood. In neural progenitor cells, MSI1 translationally regulates mammalian Numb expression by interacting with its mRNA [15]. As CML progresses to blast crisis, MSI2 is up-regulated but Numb is down-regulated. Similarly, up-regulation of MSI2 can suppress Numb expression and promote CML-blast crisis in the murine model system [16]. Based on above studies, we intend to investigate coordinate function of MSI2-Numb in the aggressive biology of PC, which has not been reported in solid tumors.

## RESULTS

### Differential expression of MSI2 and Numb expressions in PC

**Figure 1:**
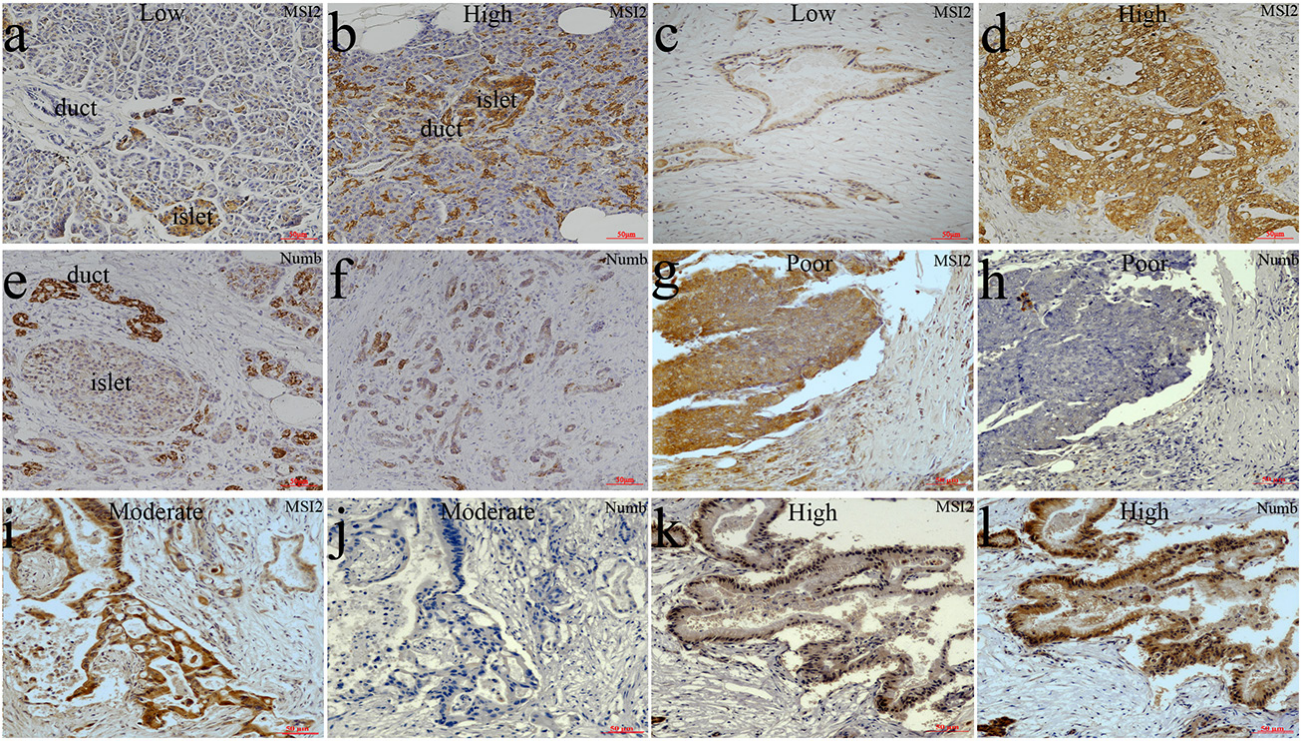
Differential expression of MSI2 and Numb in PC and paired normal pancreas in IHC assay **a, b**. Low (a) and high (b) MSI2 expressions in normal pancreatic tissues, respectively. **c, d**. Low (c) and high (d) MSI2 expressions in PC tissues, respectively. **e, f**. Numb expression in normal pancreatic tissues (e) and PC (f), respectively. **g, h**. High MSI2 (g) and negative Numb (h) expressions in the same one sample of poorly differentiated PC tissue, respectively. **i, j**. High MSI2 (i) and negative Numb (j) expressions in the same one sample of moderately differentiated PC tissue, respectively. **k, l**. Low MSI2 (k) and positive Numb (l) expressions in the same one sample of well differentiated PC tissue, respectively. (x20 magnification). Well: well differentiation; Moderate: moderate differentiation; Poor: poor differentiation.

As described previously [7, 21, 22], the location of MSI2 in cytoplasm and nuclei and Numb in membrane and cytoplasm was considered for scoring (Figure 1). IHC showed that MSI2 was overexpressed in 47 cases of total 75 PC tissues (47/75), which was much higher than that in paired adjacent normal pancreas (24/75) (*P*=0.002) (Figure 1a–1d). But no significant difference of Numb expression was observed in PC and paired adjacent pancreas (*P*>0.05). Spearman correlation tests showed a negative relationship of MSI2 and Numb expression in 75 PC tissues (*P*=0.018) (Table 1). Using serial sections, poorly and moderately differentiated PC tissues with high MSI2 expression was associated with negative Numb expression (Figure 1g–1j), whereas well differentiated PC tissues with low MSI2 expression had positive Numb expression (Figure 1k, 1l). Interestingly, both MSI2 and Numb expression were found in islet cells (Figure 1a, 1b, 1e).

**Table 1:** Correlation analysis for the relationship between MSI2 and Numb

Parameters		MSI2		r	*P*
		low	high		
Numb	negative	10	30	-0.273	0.018
	positive	18	17		

**Figure 2:**
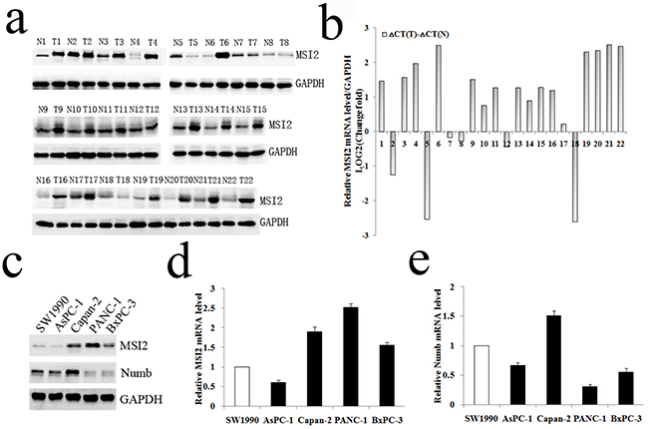
WB and qRT-PCR analysis of MSI2 and Numb expressions in PC tissues and cell lines **a, b**. WB and qRT-PCR analysis of MSI2 protein (a) and mRNA (b) levels in 22 cases of PC (T) and corresponding normal tissues (N), respectively. **c**. WB analysis of MSI2 and Numb protein levels in five PC cell lines, respectively. **d, e**. qRT-PCR analysis of MSI2 (d) and Numb (e) mRNA levels in five PC cell lines, respectively.

WB and qRT-PCR further showed that MSI2 protein and mRNA levels in 22 cases of PC tissues were much higher than that in paired adjacent pancreas, respectively (*P* <0.01; *P*=0.017) (Figure 2a, 2b). In cell level, MSI2 protein and mRNA levels were higher in Capan-2, PANC-1 and BxPC-3 cells than that in SW1990 and AsPC-1 cells, which was completely contrary to the protein and mRNA levels of Numb in these five PC cell lines (Figure 2c–2e). Taking together, a negative relationship of MSI2 and Numb was observed in both PC tissue and cell levels. Because MSI2 contained two transcript variants (NM_138962; NM_170721), the Musashi2 antibody (ab76148, Abcam) identified two isoforms in PC by WB. These results were consistent with the studies in medulloblastoma and embryonic stem cells [6, 11]. Interestingly, two isoforms corresponding bands were equally presented in cell level but unequally presented in tissue level. This inconsistence might be due to different contexts of cell and tissue micro environment, respectively.

### Association of MSI2 and Numb expressions with clinical data

Association of MSI2 and Numb expressions with clinical data was summarized in Table 2. Particularly, high MSI2 expression was positively associated with tumor size and UICC stage (*P*=0.004 and *P*=0.043, respectively), whereas Numb expression was negatively associated with tumor size, differentiation and UICC stage, respectively (*P*=0.009; *P*=0.002 and *P*=0.010, respectively) (Table 2).

**Table 2:** Association of MSI2 and Numb expressions with clinical data

Parameters	No. of patients	MSI2		*P*	Numb		*P*
		low	high		Negative	Positive	
Cases	75	28	47		40	35	
Age(years)							
≤65	55	21	34	0.801	29	26	0.861
>65	20	7	13		11	9	
Gender							
Male	52	21	31	0.411	30	22	0.255
Female	23	7	16		10	13	
Tumor location							
Head	54	21	33	0.655	28	26	0.680
Body-tail	21	7	14		12	9	
Tumor size(cm)							
<2.5	25	15	10	0.004	8	17	0.009
≥2.5	50	13	37		32	18	
Differentiation							
Well	27	13	14	0.146	8	19	0.002
Moderate and poor	48	15	33		32	16	
T stage							
T1+T2	21	11	10	0.093	9	12	0.257
T3+T4	54	17	37		31	23	
Lymph nodes metastasis							
N0(negative)	55	23	32	0.183	26	29	0.081
N1(positive)	20	5	15		14	6	
UICC stage							
I+IIA	51	23	28	0.043	22	29	0.010
IIB+III	24	5	19		18	6	
Perineural invasion							
Absent	61	25	36	0.172	31	30	0.362
Present	14	3	11		9	5	
Vascular permeation							
Absent	42	18	24	0.265	20	22	0.263
Present	33	10	23		20	13	
Pre-therapeutic CA19-9 level							
<37 U/ml	23	10	13	0.464	9	14	0.101
≥37 U/ml	52	18	34		31	21	
Postoperative Liver metastasis							
Negative	48	20	28	0.301	24	24	0.440
Positive	27	8	19		16	11	

### Association of MSI2 and Numb expressions with survival of PC patients

**Figure 3:**
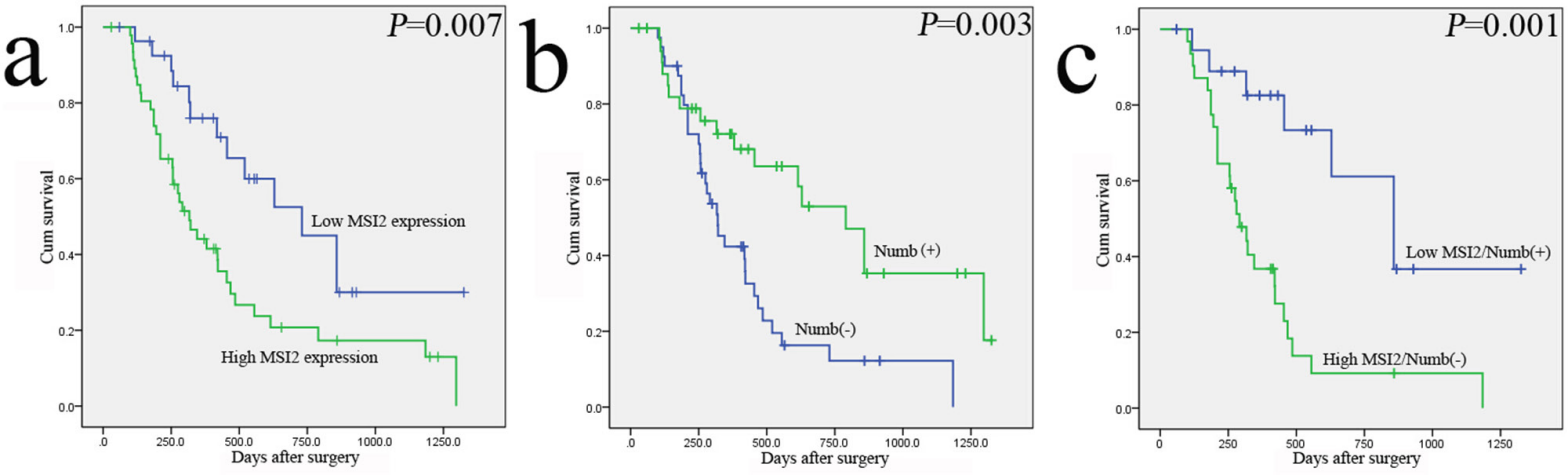
The relationship of MSI2 and Numb with the survival of 75 postoperative PC patients in Kaplan-Meier analysis **a**. High and low expression of MSI2 was plotted against overall survival time. **b**. Positive (+) and negative (-) expression of Numb was plotted against overall survival time. **c**. The negative relationship of MSI2 and Numb was plotted against overall survival time.

In Table 3, PC patients with high MSI2 expression had a significantly worse overall survival than patients with its low expression (*P*=0.007), whereas patients with Numb positive expression had a better overall survival (*P*=0.003) (Figure 3a, 3b). Moreover, high MSI2 expression combining with negative Numb expression had a significantly worse overall survival than patients with low MSI2 and positive Numb expressions (*P*=0.001) (Figure 3c). Indeed, we just add another 15 cases of PC samples based on the original 60 cases in our previous study [14]. Thus, the current kaplan-meier curve with Numb is similar to our previous survival analysis. In addition, univariate analysis showed clinicopathological factors, such as UICC stage (*P*=0.031) and postoperative liver metastasis (*P*=0.001) were also associated with patients’ prognosis. In multivariate model, MSI2 and Numb expressions and postoperative liver metastasis were independent prognostic indicators in PC patients (*P*=0.045; *P*=0.024 and *P*=0.013, respectively) (Table 3).

**Table 3:** Univariate and multivariate analysis of clinicopathological factors for survival in 75 postoperative PC patients

Parameters	Median survival (days)	Univariate analysis *P* (log rank)	Multivariate analysis hazard ratio (95% CI)	*P*
Age (<65/≥65 years)	455/420	0.177		
Gender (male/female)	454/320	0.560		
Tumor location (Head/Body-tail)	455/316	0.123		
Tumor size (<2.5/≥2.5 cm)	790/418	0.093		
well/poor and moderate Differentiation	520/321	0.085		
T stage (T1+T2/ T3+T4)	615/380	0.161		
Lymph nodes metastasis (N0/N1)	458/291	0.070		
TNM stage (I+IIA/IIB+III)	460/280	0.031	1.328(0.731-2.412)	0.351
Perineural invasion (absent/present)	421/418	0.443		
Vascular permeation (absent/present)	468/320	0.089		
CA19-9 level (<37 U/ml/ ≥37 U/ml)	468/380	0.158		
Postoperative Liver metastasis (negative /positive)	555/291	0.001	2.241(1.185-4.237)	0.013
MSI2 expression (positive/negative)	317/730	0.007	1.985(1.016-3.880)	0.045
Numb expression (positive/negative)	790/320	0.003	0.456(0.231-0.901)	0.024

### The relationship of MSI2 and Numb in PC cell lines

**Figure 4:**
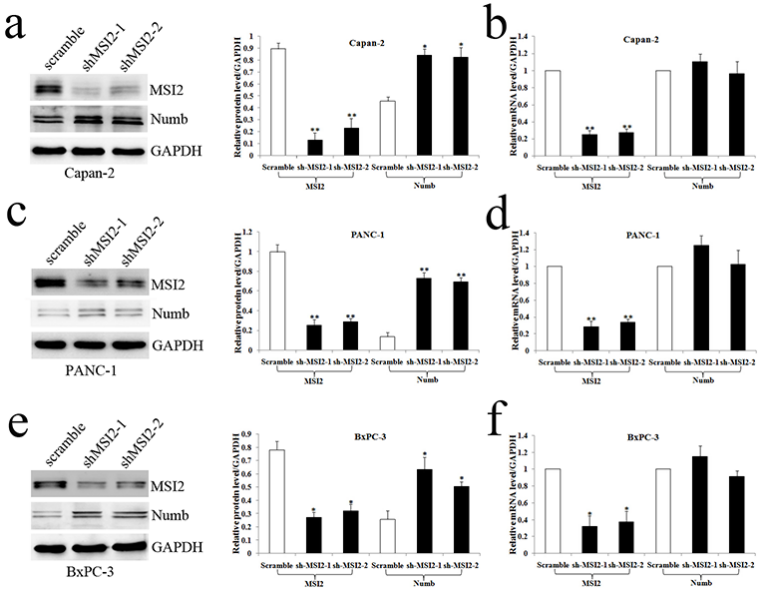
MSI2 silence up-regulated Numb protein but not mRNA level in three PC cell lines **a, b**. The protein (a) and mRNA (b) levels of MSI2 and Numb in Capan-2 cells transfected with shMSI2-1, shMSI2-2 and scramble. **c, d**. The protein and mRNA levels of MSI2 and Numb in PANC-1 cells transfected with shMSI2-1, shMSI2-2 and scramble. **e, f**. The protein and mRNA levels of MSI2 and Numb in BxPC-3 cells transfected with shMSI2-1, shMSI2-2 and scramble. Bars indicate ± S.E.*, *P* <0.05; **, *P* <0.01 compared with the control.

**Figure 5:**
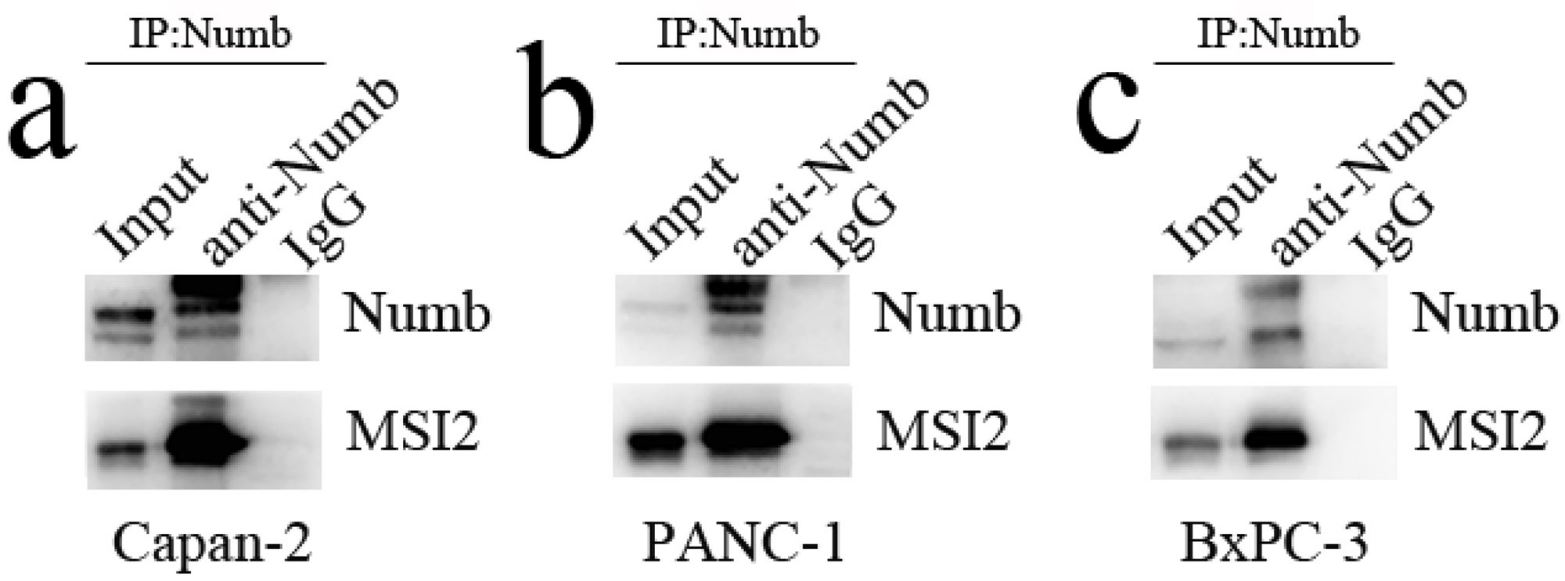
The protein interaction between MSI2 and Numb **a, b, c**. the two endogenous proteins MSI2 and Numb can be co-immunoprecipitated from cellular lysates of normal Capan-2 (a), PANC-1 (b) and BxPC-3 (c) cell lines, respectively.

Capan-2, PANC-1 and BxPC-3 cells with high MSI2 expression were used to construct MSI2 silencing stable cell lines. The protein and mRNA levels of MSI2 in these three cells with shMSI2-1 and shMSI2-2 were significantly lower than that in corresponding scramble groups (Figure 4a–4f). Meanwhile, Numb protein level was much higher in these three PC cells with shMSI2-1 and shMSI2-2 than that in scramble groups (Figure 4a, 4c, 4e). But its mRNA level in above groups showed no significant difference (Figure 4d–4f). Further co-imunoprecipitation showed that these two endogenous proteins can be co-immunoprecipitated from cellular lysates of normal Capan-2, PANC-1 and BxPC-3 cell lines (Figure 5), indicating a close protein interaction between MSI2 and Numb. Because less protein was loaded in the input lanes, the bands in input groups seem much lower than that in anti-Numb groups (Figure 5).

### Coordinate regulation of MSI2 and Numb in cell invasion and migration of PC cells

**Figure 6:**
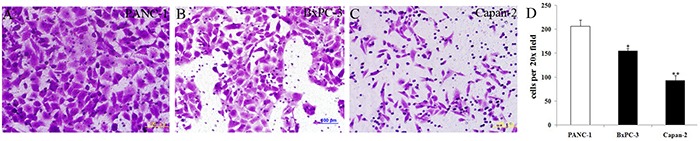
Cell invasion in three normal PC cell lines with the same cell intensity **a–d**. With the same cell intensity of three PC cells, cell invasion was high in PANC-1 (a), moderate in BxPC-3 (b), and low in Capan-2 (c) cell lines, respectively. Bars indicate ± S.E.*, *P* <0.05; **, *P* <0.01 compared with the control.

Under the same cell intensity, cell invasion was high in PANC-1 cells, moderate in BxPC-3 cells and low in Capan-2 cells (Figure 6), which was corresponding to the decreasing MSI2 and increasing Numb expression in these three PC cell lines, respectively (Figure 2 and Figure 6). It indicated that high MSI2 and low Numb levels were closely related to the strong invasion of PC cells.

**Figure 7:**
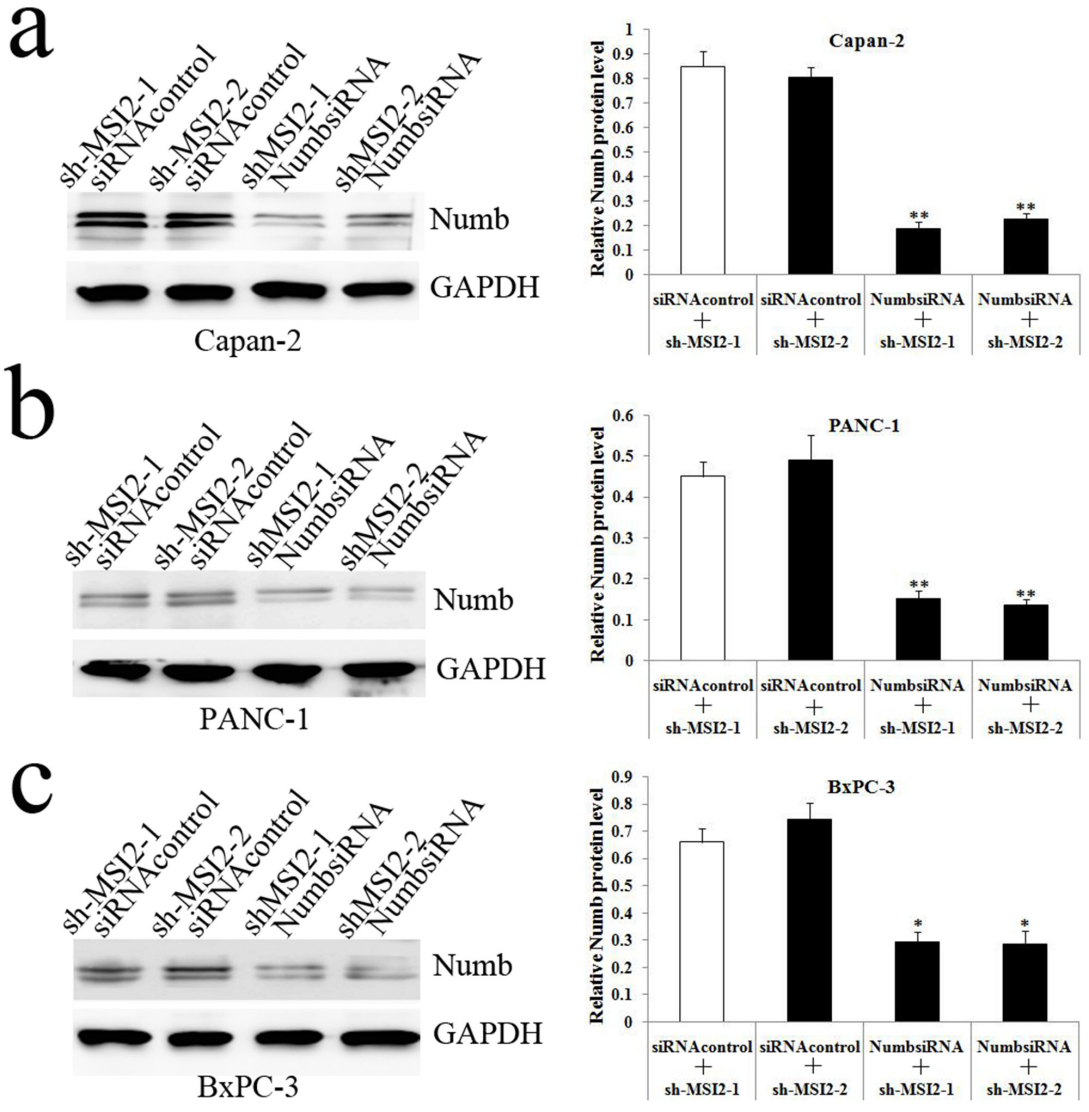
NumbsiRNA can significantly repress the up-regulation of Numb protein induced by MSI2 silence in PC cell lines **a, b, c**. NumbsiRNA can significantly repress the up-regulation of Numb protein in shMSI2-1 and shMSI2-2 transfected Capan-2 (a), PANC-1 (b) and BxPC-3 (c) cell lines, respecitively. Bars indicate ± S.E.*, *P* <0.05; **, *P* <0.01 compared with the control.

Next, Capan-2, PANC-1 and BxPC-3 cells with shMSI2-1 and shMSi2-2 were transfected with NumbsiRNA or siRNA control, respectively. WB showed that Numb knockdown can significantly repress the increase of Numb protein induced by MSI2 silence in these three cell lines (Figure 7).

**Figure 8:**
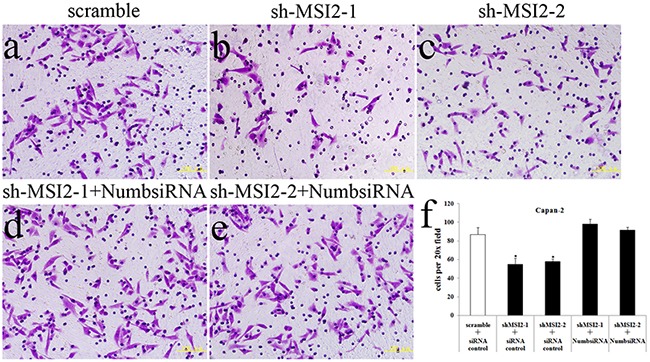
Coordinate regulation of MSI2 and Numb in cell invasion of Capan-2 cells **a–f**. Cell invasion in shMSI2-1 (b) and shMSI2-2 (c) transfected Capan-2 cells was significantly decreased, compared with that in corresponding scramble group (a). However, Numb knockdown can significantly reverse the decrease of cell invasion in shMSI2-1 (d) and shMSI2-2 (e) transfected Capan-2 cells. Bars indicate ± S.E.*, *P* <0.05 compared with the control.

**Figure 9:**
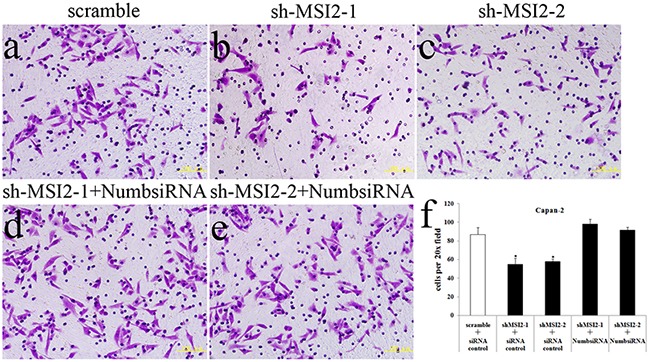
Coordinate regulation of MSI2 and Numb in cell invasion of PANC-1 cells **a–f**. Cell invasion in shMSI2-1 (b) and shMSI2-2 (c) transfected PANC-1 was significantly decreased, compared with that in corresponding scramble group (a). However, Numb knockdown can significantly reverse the decrease of cell invasion in shMSI2-1 (d) and shMSI2-2 (e) transfected PANC-1 cells. Bars indicate ± S.E.*, *P* <0.05 compared with the control.

**Figure 10:**
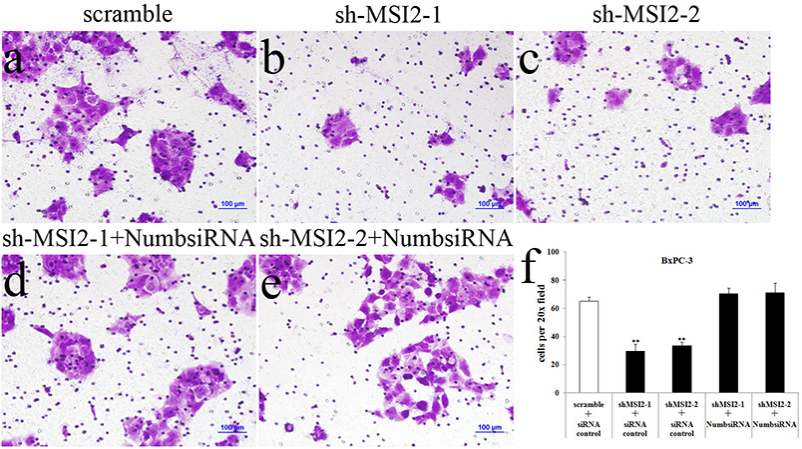
Coordinate regulation of MSI2 and Numb in cell invasion of BxPC-3 cells **a–f**. Cell invasion in shMSI2-1 (b) and shMSI2-2 (c) transfected BxPC-3 was significantly decreased, compared with that in corresponding scramble group (a). However, Numb knockdown can significantly reverse the decrease of cell invasion in shMSI2-1 (d) and shMSI2-2 (e) transfected BxPC-3 cells. Bars indicate ± S.E.*, *P* <0.05; **, *P* <0.01 compared with the control.

Cell invasion in shMSI2-1 and shMSI2-2 transfetced Capan-2, PANC-1 and BxPC-3 cells was significantly decreased, compared with that in corresponding scramble groups. However, Numb knockdown can significantly reverse the decrease of cell invasion induced by MSI2 silence in these three PC cell lines (Figure 8, 9, 10).

**Figure 11:**
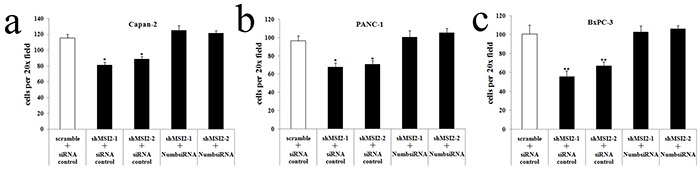
Coordinate regulation of MSI2 and Numb in cell migration of three PC cell lines **a, b, c**. Cell migration in shMSI2-1 and shMSI2-2 transfetced Capan-2 (a), BxPC-3 (b) and PANC-1 (c) cells was significantly decreased, compared with that in corresponding scramble groups. However, Numb knockdown can significantly reverse the decrease of cell migration in shMSI2-1 and shMSI2-2 transfetced PC cell lines (a, b, c), respectively. Bars indicate ± S.E.*, *P* <0.05; **, *P* <0.01 compared with the control.

In accordance with the results of cell invasion assays, MSI2 silence inhibited cell migration in Capan-2, PANC-1 and BxPC-3 cells. However, Numb knockdown can significantly reverse the decrease of cell migration induced by MSI2 silence in these three PC cell lines (Figure 11). Taking together, MSI2 promotes invasion and migration of PC cells by down-regulating Numb protein.

### MSI2 silencing inhibited subcutaneous tumors formation and distant liver metastasis of pancreatic tumor in nude mice

**Figure 12:**
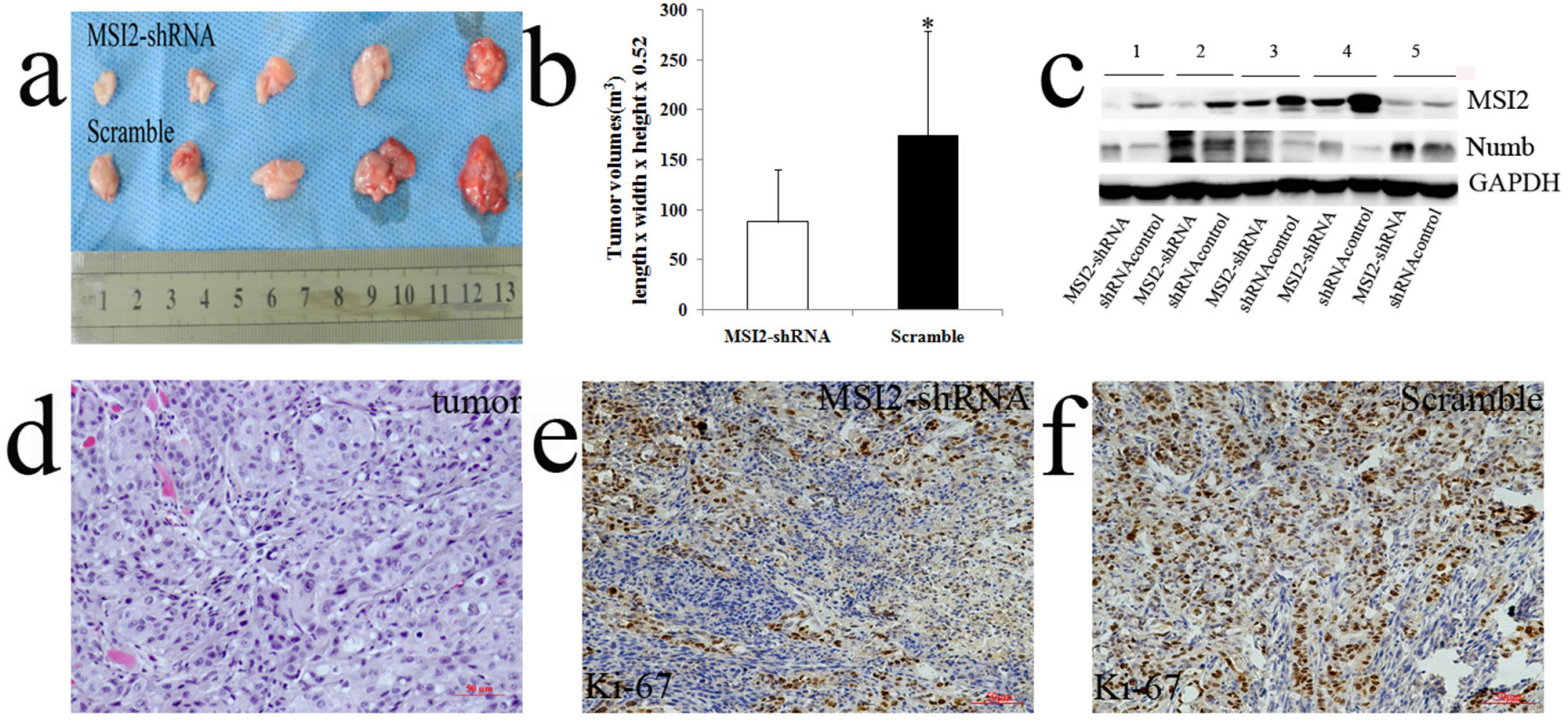
MSI2 silence inhibited subcutaneous tumors growth in vivo **a, b**. tumor volumes in nude mice implanted with shMSI2-1 transfected BxPC-3 cells were smaller than that in paired corresponding scramble groups. **c**. WB was performed to verify MSI2 silence in tumor samples. Numb protein in subcutaneous tumor of MSI2-shRNA group is significantly higher than that in scramble group. **d**. The primary tumors were diagnosed by histopathological examination with HE staining **e, f**. Ki-67 expression in subcutaneous tumors with shMSI2-1 (e) and scramble groups (f), respectively. Bars indicate ± S.E. *, *P* <0.05 compared with the control.

Tumor volumes in nude mice implanted with shMSI2-1 transfected BxPC-3 cells were smaller than that in paired corresponding scramble groups (*P*=0.032) (Figure 12a, 12b). WB was performed to verify MSI2 silence in the tumor samples. In line with the results in cell level, Numb protein expression in subcutaneous tumor of MSI2-shRNA groups is significantly higher than that in scramble groups (*P*<0.01) (Figure 12c). The primary tumors were diagnosed by histopathological examination under HE staining (Figure 12d). Meanwhile, Ki-67, as a key marker of proliferation in tumor progression, was detected in two groups by IHC. We showed that Ki-67 expression was significantly lower in shMSI2-1 group than that in scramble group (*P*<0.01) (Figure 12e, 12f). It indicated that MSI2 also promoted tumor growth by up-regulating Ki-67 expression in vivo.

**Figure 13:**
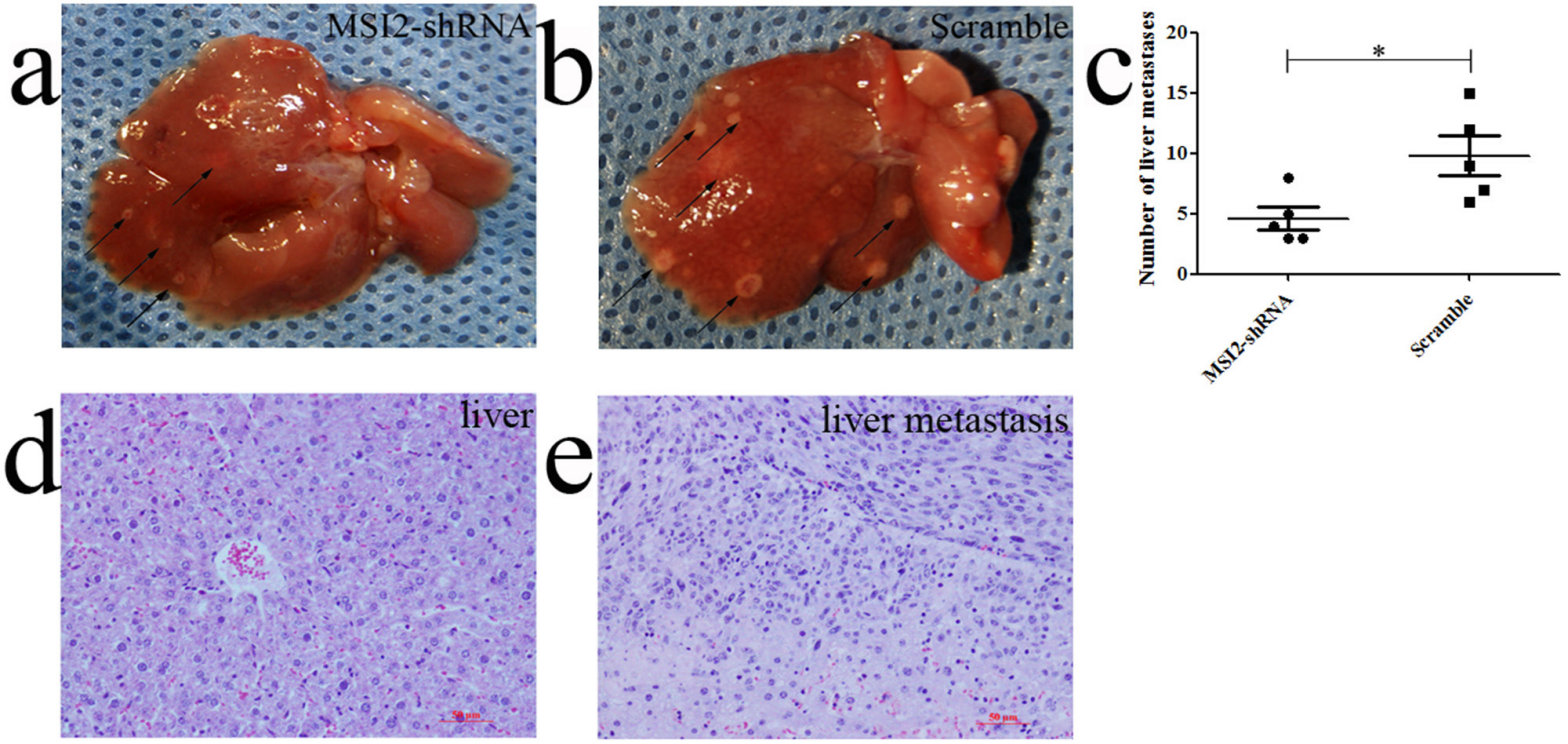
MSI2 silence inhibited the frequency of liver metastasis in vivo **a, b, c**. the average number of liver metastases in nude mice implanted with shMSI2-1 transfected PANC-1cells was much lower than that in corresponding scramble groups. **d**. The normal liver tissues were diagnosed by HE staining. **e**. The liver metastases were diagnosed by HE staining. Bars indicate ± S.E. *, *P* <0.05 compared with the control.

The average number of liver metastases in nude mice implanted with shMSI2-1 transfected PANC-1cells was much lower than that in scramble groups (*P*=0.030) (Figure 13a–13c). The distant liver metastases were diagnosed by histopathological examination under HE staining (Figure 13d, 13e). Based on invasion assays in vitro, MSI2 might promote PC metastasis in vivo.

## DISCUSSION

Previous studies show that MSI2 is overexpressed in various tumors, such as aggressive myeloid leukemia, hepatocellular and pulmonary cancers [8, 9, 23, 24]. In our study, MSI2 was also overexpressed in PC and established an inverse correlation with Numb. Meanwhile, high MSI2 expression was positively associated with tumor size and UICC stage in PC patients, whereas Numb positive expression was negatively associated with tumor size, UICC stage and differentiation. These results are consistent with the studies in hepatocellular and breast cancers [9, 21–23]. Additionally, MSI2 and Numb were independent adverse and favorable indicators for the survival of PC patients, respectively. Moreover, the negative relationship between them co-influenced PC patients’ survival, which has not been reported previously. All data above indicates that MSI2-Numb interaction sheds light on a key to understand the malignancies of PC.

The increase of MSI2 and decrease of Numb are also found in 33 samples of myeloid blast crisis by comparing with 57 chronic phase CML [16]. In 90 CML patients, a marked up-regulation of MSI2 is observed in every patient during CML progression, whereas Numb is down-regulated in a majority of blast crisis patients [8]. But no significant relationship of them is reported in acute myeloid leukemia (AML) and hepatocellular cancer [9, 25, 26]. In addition to above controversy, MSI2 predicts unfavorable outcome in AML and hepatocellular cancer [7, 9, 23, 26], whereas Numb is a better prognostic markers in breast cancer and salivary gland carcinomas [21, 22, 27]. However, Thol et al. show that only MSI2 but not Numb is associated with shorter overall survival in 454 AML patients [25]. The differences of above studies are questionable in terms of different cancer types, sample size and statistical methods as Stocken et al suggest [28]. Taking together, the clinicopathological significance of MSI2 and Numb in PC patients drives us to further investigate their interaction in vitro and vivo.

In our study, the negative expression of MSI2 and Numb was also observed in five PC cell lines. Meanwhile, MSI2 silence up-regulated Numb protein level in vitro and vivo, which has been rarely reported in solid tumors. Only one study in medulloblastoma shows that MSI2 knockdown significantly up-regulates Numb protein in DAOY MB cells [11]. But MSI2-Numb interaction was ubiquitously observed in myeloid leukemia. MSI2 knockdown increases Numb protein in both AML THP-1cells and CML LAMA-84 cells. Conversely, MSI2 overexpression in chronic phase CML cells leads to down-regulation of Numb [8, 16]. Interestingly, the mRNA level of Numb wasn't altered in MSI2 silencing PC cells. It suggests that MSI2 down-regulates Numb at the translational level. However, the corresponding mechanism of MSI2-Numb interaction remains unclear. Our study first demonstrated that these two endogenous proteins can be co-immunoprecipitated in PC cell lines, which offers a novel direction to investigate their relationship in protein level.

Finally, MSI2 silence decreased cell invasion and migration in vitro and inhibited the growth of primary tumors and the number of liver metastases in vivo. The similar results are also observed in aggressive myeloid leukemia and hepatocellular cancer [8, 9, 16, 23]. Moreover, our study first showed that Numb knockdown can significantly reverse the decrease of PC invasion and migration induced by MSI2 silence. In myeloid leukemia, high Numb expression or MSI2 silence could make leukemia more differentiated and unable to propagate disease markedly. Conversely, NUP98-HOXA9, as a genetic event in blast crisis CML, up-regulates MSI2 which in turn post-transcriptionally inhibits Numb expression, and finally results in propagation and progression of the aggressive state [8, 29, 30]. Although we do not verify the suppressive role of Numb in MSI2 oncogenic function in vivo, their close interaction is proved in vitro and vivo in current study. Meanwhile, the growth of tumor formation in nude mice implanted with Numb isoform-1 overexpressing esophageal squamous cancer cells is significantly inhibited [31]. Taking together, MSI2 might promote PC metastasis by down-regulating Numb.

In conclusion, Musashi2 promotes the development and progression of PC by down-regulating Numb protein. In mammals, MSI1 activates Notch signaling through the translational repression of Numb which inhibits intracellular Notch signaling [5]. Numb also prevents ubiquitination and degradation of p53 by inactivating MDM2 and suppresses Hedgehog signaling by targeting Gli1 [13, 32]. Our future study will focus on the interaction of MSI2-Numb-Notch, MSI2-Numb-p53 and MSI2-Numb-Gli1 in PC development. In addition, recent study reports that SOX2 knockdown and overexpression inhibits and promotes cell proliferation in PC cells, respectively [33]. In medulloblastoma, MSI2 is a typical SOX2-associated protein [11]. Whether MSI2-Numb interaction is regulated by SOX2 in PC is a promising target for us in the future.

## MATERIALS AND METHODS

### Tissue samples

All patient-derived specimens were collected and archived under protocols approved by the institutional review board of China Medical University. Seventy-five cases of paraffin-embedded pancreatic ductal adenocarcinoma and paired adjacent normal specimens were obtained from patients at the First Hospital of China Medical University between 2006 and 2014. All diagnoses were confirmed pathologically. Patients with endocrine carcinoma, acinar cell carcinoma and invasive intraductal papillary mucinous carcinoma were excluded from this study. The histologically normal tissues were at least 2 cm away from the cancer. Moreover, additional 22 paired fresh PC and adjacent normal pancreas were prepared for late protein and mRNA extraction. A dedicated table for patients’ characteristics was summarized in Supplementary Table S1. As previously described [17], postoperative patients regularly underwent laboratory examinations, including tumor markers, liver function, US, abdominal CT/PET or contrast MRI. If the liver metastasis showed no definite evidence of other metastasis or recurrence elsewhere, we characterized the newly developed hepatic lesion as postoperative liver metastasis,

### Cell lines and culture

AsPC-1, BxPC-3, PANC-1, and SW1990 human PC cell lines were purchased from the Cell Bank of the Chinese Academy of Sciences (Shanghai, China). Capan-2 cells were obtained from the American Type Culture Collection (Manassas, VA, USA). These cell lines were maintained in the recommended growth media with 10% fetal calf serum (Hyclone, Logan, UT, USA).

### Immunohistochemistry

Immunohistochemistry (IHC) was performed as described previously [14, 18]. Staining intensity was scored as 0 (negative), 1 (weak), 2 (medium), and 3 (strong). Extent of staining was scored as 0 (0%), 1 (1–25%), 2 (26–50%), 3 (51–75%), and 4 (76–100%) according to the percentage of the entire carcinoma area that was positively stained. The final IHC staining scores were determined by three professional pathologists at our hospital. In normal pancreas, only the non-neoplastic pancreatic ductal cells were scored. The acinar and islet cells were not included in the scoring. The sum of the extent and intensity score was used as the final staining scores (0–7). For MSI2, tumors with a final staining score of >3.5 were considered as high expression. For Numb, tumors with a final staining score of ≥2 were considered as positive expression.

### Western blot

For Western Blot (WB), whole protein lysates were prepared from PC tissues and cell lines. Samples were loaded onto 10% SDS-polyacrylamide gels, transferred to polyvinylidene difluoride membranes (Millipore Corp, Bedford, MA, USA) and incubated with primary MSI2 (Abcam, Cambridge, UK) and Numb (Cell signaling technology, MA, USA) antibodies overnight at 4°C. Membranes were incubated with horseradish peroxidase-conjugated monoclonal secondary antibody (Santa Cruz, CA, UK) at room temperature for 1.5 hr, respectively. Immunoreactive protein bands were visualized with an ECL detection kit (ECL, Thermol Biotech Inc, USA). Each experiment was repeated three times.

### qRT-PCR

As described previously [19], total RNA was extracted from tissue samples and PC cells with TRIZOL reagent under the manufacturer (Takara Bio, Otsu, Japan). We keep equal quantity for RNA level in all samples via nucleotide determination before qRT-PCR detection. cDNA was synthesized from total RNA using the Expand Reverse Transcriptase Kit (Thermo Biotech Inc, USA). The expression of MSI2 and Numb was analyzed in a Light Cycler 2.0 with the Light Cycler kit (Takara). The conditions were as follows: 95°C for 30s, 40 cycles of 95°C for 5s and 60°C for 30s. DEPC water was used in place of the template cDNA for the negative control. The primers were as follows: MSI2, 5’ - AGACCTCACCAGATAGCCTTAG -3’ (sense) and 5’ - CCACTACTGTGTTCGCAGATAA -3’ (antisense); Numb, 5’ - CACTCGTCGCTGGATCTGTCA - 3’ (sense) and 5’ - CACAGCCTACTGCATGGCTCA -3’ (antisense); GADPH, 5’ - CATGAGAAGTATGACAACAGCCT -3’ (sense) and 5’ - AGTCCTTCCACGATACCAAAGT -3’ (antisense). The quality of the PCR products was determined by post-PCR melt-curve analysis. The expression level was calculated using the 2^-△△Ct^ method (relative quantification). Each experiment was repeated three times.

### Construction of stable cell lines and RNA interference

Two shRNA plasmids of MSI2 were kindly offered by Hope KJ et al as described previously [20]. The plasmids of MSI2-shRNA and shRNA control were successfully packaged into Lentivector (LV) (GenePharma Co, Ltd, Shanghai, China). LV3-H1-MSI2-shRNA and LV3-H1-shRNA-control were represented for shMSI2-1/shMSI2-2 and scramble, respectively. PC cells were transfected with shMSI2-1/shMSI2-2 and scramble for 24hrs. Puromycin was used to screen the transfected cells for 48hrs twice. MSI2 silencing effect was successfully verified by WB and qRT-PCR (Figure 4). Finally, LV transfected PC cells were quickly collected for late experiments in order to avoid excessive cell passages before in vitro and in vivo assays. The interference effect for NumbsiRNA with three target sequences was effectively verified in our previous study [14]. NumbsiRNA and siRNA control were synthesized from GenePharma Company (GenePharma Co, Ltd). Cells were transiently transfected with siRNA (20 uM) for 48-72 hrs using oligofectamine (Invitrogen, Carlsbad, CA, USA) as described by the manufacturer. All target sequences mentioned above were shown in Supplementary Table S2.

### Co-immunoprecipitation

As described previously [14], Capan-2, PANC-1 and BxPC-3 PC cells were extracted in a lysis buffer (20 mM Tris/HCl, pH7.4, 1.0% NP-40, 1 mM EDTA, 150 mM NaCl, 50 μg/ml PMSF, 10 μg/ml leupeptin). The soluble supernatants were incubated with the indicated Numb antibody overnight at 4°C. The immunocomplexes were then precipitated with protein A-Sepharose 4B (Sigma, St. Louis, MO, USA), washed three times with lysis buffer, eluted by boiling in sample buffer for SDS–PAGE, and then subjected to IB analysis with the MSI2 antibody. Each experiment was repeated three times.

### Invasion and migration assays

Cell invasion and migration were detected with modified Boyden chamber (BD Biosciences, Sparks, MD, USA) assays. Briefly, three PC cell lines were seeded onto 8.0-µM pore size membrane inserts with 8.0 µM pores coated with matrigel (BD Biosciences) in 24-well plates with FBS-free growth media. 1640 plus 10% FBS was added to the bottom of the wells as a chemoattractant. 12-24 hrs later, cells that did not migrate were removed from the top side of the inserts with a cotton swab. Cells that had migrated to the underside of the inserts were stained with Crystal Violet Hydrate (Sigma) according to the manufacturer's instruction. The migratory cells were counted under a microscope at 20 x magnification. Cell images were obtained using a microscope (Nikon Microphot-FX, Japan), and counted in five random fields per insert. The migration assay was done in a similar fashion without matrigel. In more detail, we first used the same cell intensity (5×10^5/ml) to investigate different invasion ability of three normal PC cell lines. Because too high or too low cell population in invasion and migration assay is unfavorable for late result analysis in our pre-experiment, we seeded 1×10^5/ml of PANC-1 cells, 2.5×10^5/ml of BxPC-3 cells and 5×10^5/ml of Capan-2 cells in cell invasion and migration assayt. The results are presented as cells (actual number) migrated per field.

### In vivo xenograft model

Animals were maintained according to institutional regulations in facilities approved by the Animal Care Committee of China Medical University in accordance with Chinese government guidelines for animal experiments.

Bilateral axillas injected manner is used to construct subcutaneous tumor formation, while tail vein injected manner is used to construct blood metastasis (liver metastasis). In our pre-experiment, BxPC-3and PANC-1 cells were easy for constructing above xenograft models.

Total 15 cases of nude mice were acclimatized for a week. shMSI2-1 and scramble transfected BxPC-3 cells (1×10^6/L) suspended in 100ul of FBS-free 1640 were subcutaneously transplanted into bilateral axillas of 5 cases of mice, respectively. A cotton swab was used to avoid possible bleeding and leakage of tumor cells from the injection site. The mice were sacrificed 4 weeks later. Tumors were excised and were documented by measurements using vernier calipers. Tumor volumes were calculated by the following formula: length x width x height x 0.52 in millimeters. Finally, tumor samples were extracted for WB analysis and fixed for late hematoxylin and eosin (HE) and IHC staining.

shMSI2-1 and scramble transfected PANC-1 cells (1×10^7) suspended in 100ul of FBS-free DMEM were injected into the tail vein of 10 cases of nude mice, respectively. A cotton swab was held over the injection site for 1 min to prevent leakage from tail vein. The mice were sacrificed 6-8 weeks later. The number of liver metastases was investigated immediately, and then fixed for HE staining.

### Statistical analysis

Statistical analyses were performed using SPSS software 13.0 (SPSS, Chicago, IL, USA). The clinicopathological significance of and relationship between MSI2 and Numb expressions were analyzed using Chi-squared and Spearman correlation tests, respectively. The Kaplan-Meier method was used to estimate survival, and differences were analyzed by the log-rank test. Cox's proportional hazards regression model in a stepwise manner was used to analyze independent prognostic factors. The differences of MSI2 and Numb expression in PC and paired adjacent pancreas and of orthotopic tumor volumes in bilateral axillas were compared through Paired sample t-test. The differences of cell migration and invasion assays and of the number of liver metastases were compared through Student's t-test. *P*<0.05 was considered to be statistically significant.

This work was supported by Scientific Research of Special-Term Professor from the Educational Department of Liaoning Province, China (Liao Cai Zhi Jiao No. 2012-512) and the Social Development Program from Shenyang Science and Technology Bureau, China (No. F15-139-9-19).

## SUPPLEMENTARY TABLES


