# Correction: Musashi2 promotes the development and progression of pancreatic cancer by down-regulating Numb protein

**DOI:** 10.18632/oncotarget.28157

**Published:** 2022-01-20

**Authors:** Weiwei Sheng, Ming Dong, Chuanping Chen, Yang Li, Qingfeng Liu, Qi Dong

**Affiliations:** ^1^Department of General Surgery, Gastrointestinal Surgery, The First Hospital, China Medical University, Shenyang, 110001, China; ^2^Department of Clinical Laboratory, The Sixth Peoples’ Hospital of Shenyang City, 110003, China; ^3^Department of Cell Biology, China Medical University, Shenyang, 110001, China; ^4^Department of General Surgery, The Peoples’ Hospital of Liaoning Province, Shenyang, 110015, China

**This article has been corrected:** In Figure 9, panels ‘B’ and ‘C’ contain an accidental overlap. The corrected Figure 9, produced using the original data, is shown below. The authors declare that these corrections do not change the results or conclusions of this paper.

Original article: Oncotarget. 2017; 8:14359–14373. 14359-14373. https://doi.org/10.18632/oncotarget.8736

**Figure 9:**
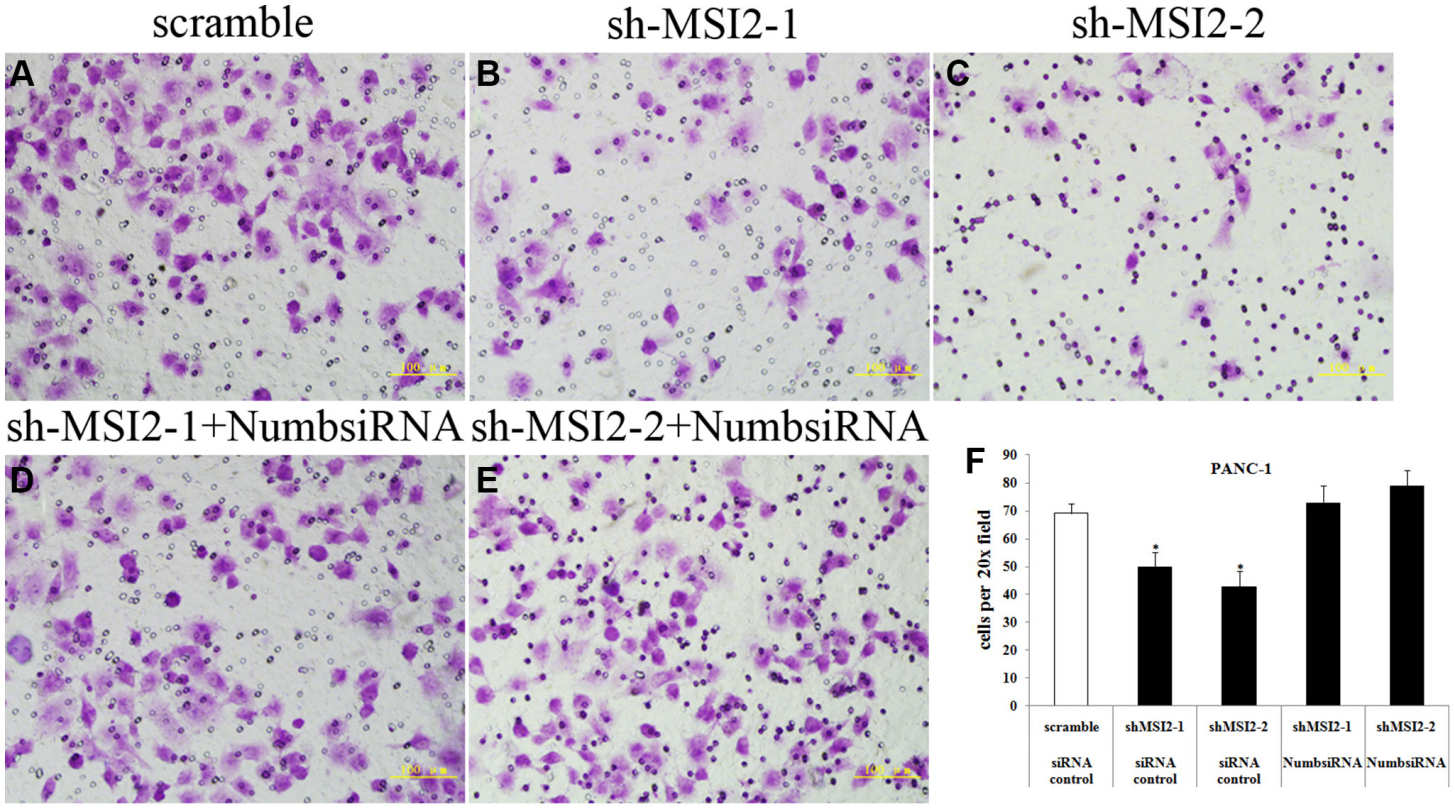
Coordinate regulation of MSI2 and Numb in cell invasion of PANC-1 cells. (**A**–**F**) Cell invasion in shMSI2-1 (B) and shMSI2-2 (C) transfected PANC-1 was significantly decreased, compared with that in corresponding scramble group (A). However, Numb knockdown can significantly reverse the decrease of cell invasion in shMSI2-1 (D) and shMSI2-2 (E) transfected PANC-1 cells. Bars indicate ± S.E. ^*^ *P* < 0.05 compared with the control.

