# Supplementary material for: Musashi2 promotes the development and progression of pancreatic cancer by down-regulating Numb protein
Source: Oncotarget. 2016 Apr 15;8(9):14359–73. doi: 10.18632/oncotarget.8736 (PMC5362411; doi:10.18632/oncotarget.8736)
Supplement: Supplementary file 1 [file oncotarget-08-14359-s001.pdf]

SUPPLEMENTARY TABLES

Supplementary Table S1: Clinical data of 75 PC patients

Parameters	No. of patients
Total Cases	75
Age(years)	
≤65	55
>65	20
Gender	
Male	52
Female	23
Tumor location	
Head	54
Body-tail	21
Tumor size(cm)	
<2.5	25
≥2.5	50
Differentiation	
Well	27
Moderate and poor	48
T stage	
T1+T2	21
T3+T4	54
Lymph nodes metastasis	
N0(negative)	55
N1(positive)	20
UICC stage	
I+IIA	51
IIB+III	24
Perineural invasion	
Absent	61
Present	14
Vascular permeation	
Absent	42
Present	33
Pre-therapeutic CA19-9 level	
<37 U/ml	23
≥37 U/ml	52
Postoperative Liver metastasis	
Negative	48
Positive	27

Supplementary Table S2: The target sequences of shMSI2-1, shMSI2-1, scramble, NumbsiRNA and siRNA control

Gene	Oligo Name	Oligo Sequence
MSI2	shMSI2-1	TGCTGTTGACAGTGAGCGACCGGATTTGCTCCTAGCTATGTA GTGAAGCCACAGATGTACATAGCTAGGAGCAAATCCGGGT GCCTACTGCCTCGGA
	shMSI2-1	TGCTGTTGACAGTGAGCGCGCAAGTGTAGATAAAGTA TTATAGTGAAGCCACAGATGTATAATACTTTATCTACACT TGCTTGCCTACTGCCTCGGA
NA	Scramble	TGCTGTTGACAGTGAGCGCAAGGTTAAGTCGCCCTCGCTC TAGTGAAGCCACAGATGTAGAGCGAGGGCGACTTAA CCTTATGCCTACTGCCTCGGA
Numb	siRNA1	Sense: 5′ CUGGAAAGAAAGCAGUUAATT 3′
		Antisense: 5′ UUAACUGCUUUCUUUCCAGTT 3′
	siRNA2	Sense: 5′ GAAGAUGUCACCCUUUAAATT 3′
		Antisense: 5′ UUUAAAGGGUGACAUCUUCTT 3′
	siRNA3	Sense: 5′ GGAUCUGUCACUGCUUCAUTT 3′
		Antisense: 5′ AUGAAGCAGUGACAGAUCCTT 3′
NC	siRNA control	Sense: 5‘ UUCUCCGAACGUGUCACGUTT 3’
		Antisense: 5‘ UUCUCCGAACGUGUCACGUTT 3’
